# Obesity with Comorbid Eating Disorders: Associated Health Risks and Treatment Approaches

**DOI:** 10.3390/nu10070829

**Published:** 2018-06-27

**Authors:** Felipe Q. da Luz, Phillipa Hay, Stephen Touyz, Amanda Sainsbury

**Affiliations:** 1 The Boden Institute of Obesity, Nutrition, Exercise & Eating Disorders, Faculty of Medicine and Health, Charles Perkins Centre, The University of Sydney, Camperdown, NSW 2006, Australia; Amanda.salis@sydney.edu.au; 2 Faculty of Science, School of Psychology, the University of Sydney, Camperdown, NSW 2006, Australia; Stephen.touyz@sydney.edu.au; 3 CAPES Foundation, Ministry of Education of Brazil, Brasília, DF 70040-020, Brazil; 4 Translational Health Research Institute (THRI), School of Medicine, Western Sydney University, Locked Bag 1797, Penrith, NSW 2751, Australia; P.hay@westernsydney.edu.au

**Keywords:** obesity, eating disorders, binge eating, dieting, treatment

## Abstract

Obesity and eating disorders are each associated with severe physical and mental health consequences, and individuals with obesity as well as comorbid eating disorders are at higher risk of these than individuals with either condition alone. Moreover, obesity can contribute to eating disorder behaviors and vice-versa. Here, we comment on the health complications and treatment options for individuals with obesity and comorbid eating disorder behaviors. It appears that in order to improve the healthcare provided to these individuals, there is a need for greater exchange of experiences and specialized knowledge between healthcare professionals working in the obesity field with those working in the field of eating disorders, and vice-versa. Additionally, nutritional and/or behavioral interventions simultaneously addressing weight management and reduction of eating disorder behaviors in individuals with obesity and comorbid eating disorders may be required. Future research investigating the effects of integrated medical, psychological and nutritional treatment programs addressing weight management and eating disorder psychopathology in individuals with obesity and comorbid eating disorder behaviors—such as binge eating—is necessary.

## 1. Introduction

Obesity is commonly associated with health complications. Individuals with obesity are at high risk of several physical diseases, such as certain cancers, diabetes, hypertension, heart disease, stroke, as well as being at increased risk of mortality [1,2,3,4,5,6,7]. Obesity is also often associated with mental health problems and psychosocial difficulties. Indeed, women with obesity tend to report worse mental health than women without obesity [8]. Moreover, women tend to experience more mental health complications associated with obesity than men [9], albeit mental health problems associated with obesity (e.g., anxiety) occur in both women and men [9]. One factor that can be detrimental to the mental health of individuals with obesity is exposure to well-documented discriminatory attitudes and behaviors in different areas, such as employment, education and healthcare [10]. Additionally, discrimination against individuals with higher body mass index (BMI) can be particularly problematic because it can induce strong dissatisfaction with one’s body weight and/or shape, which is a risk factor for the development of comorbid eating disorder behaviors [11].

The eating disorders that have been most frequently studied in individuals with obesity are binge eating disorder and bulimia nervosa. Binge eating disorder is defined in the current Diagnostic and Statistical Manual of Mental Disorders (DSM-5) by recurrent binge eating episodes occurring at least once a week for the past 3 months, and associated with marked distress [12]. The DSM-5 criteria for binge eating disorder also require that individuals experience at least three of the following five features: eating much more quickly than normal; eating until feeling excessively full; overeating when not feeling physically hungry; eating alone because of embarrassment related to the amount of food consumed; and feeling disgusted, depressed, or very guilty after the binge eating episodes [12]. Bulimia nervosa is characterized by self-evaluation that is excessively influenced by body weight and shape, recurrent binge eating, and recurrent unhealthy compensatory behaviors to prevent weight gain (i.e., self-induced vomiting; misuse of laxatives, diuretics or other medications; fasting; excessive exercise) [12]. The DSM-5 criteria for bulimia nervosa require that the binge eating episodes and compensatory unhealthy behaviors have occurred at least once per week for the past 3 months [12]. In addition to binge eating disorder and bulimia nervosa, individuals with obesity can also experience “other specified feeding or eating disorder” when presentations cause significant distress or impairment but do not completely meet the DSM-5 criteria for a specific eating disorder [12]. For example, according to the DSM-5, a person may have binge eating disorder of low frequency and/or limited duration. In this case, the person meets the DSM-5 criteria for binge eating disorder, except that binge eating has occurred less than once a week and/or for less than 3 months [12].

There is a significant co-occurrence of eating disorders, particularly binge eating disorder, in individuals with higher BMI. In Latin America, for example, the prevalence of binge eating disorder was 16–52% in individuals with obesity (BMI ≥ 30 kg/m^2^) attending weight loss programs [13]. In the United States, a study with a nationally-representative sample of 9282 people assessed in 2001–2003 found that 42% of individuals that had had a binge eating disorder at any stage in their life had obesity at the time of the survey [14]. A more recent study in the United States, with a sample of 36,306 participants assessed in 2012–2013, found that relative to those with no history of eating disorders, participants who met criteria for binge eating disorder in the last 12 months, or at any time in their lives, had significantly increased odds of having obesity or extreme obesity [15]. On the flip side, a study with a clinical sample of 1383 individuals with current eating disorders, including 123 with binge eating disorder and 551 with bulimia nervosa, found that 87% of individuals with binge eating disorder, and 33% of individuals with bulimia nervosa, had also had obesity at some point in their lives [16]. These studies show a significant co-occurrence of obesity and eating disorders, and are consistent with the hypothesis that these conditions can potentially contribute to and/or exacerbate each other. Additionally, obesity with comorbid eating disorder behaviors, such as binge eating, may be a growing problem. In a population-representative sample of 9053 people in Australia between the years of 1995 to 2015, there were significant increases in the prevalence of obesity and eating disorder behaviors independently; however, the greatest increases were in the prevalence of individuals with obesity and comorbid binge eating or very strict dieting (7.3 fold and 11.5 fold, respectively) [17]. The increases in the prevalence of obesity and comorbid eating disorder behaviors mentioned above may be related to a potential contribution of binge eating to obesity [14,18,19], as well as to the social expectancy for people with obesity to lose excess weight [17]. The potentially growing prevalence of individuals with obesity and comorbid eating disorder behaviors is concerning due to the medical and psychosocial risks that these individuals are exposed to.

## 2. Health Risks of Obesity with Comorbid Eating Disorders

Individuals with obesity and comorbid eating disorders are at high risk of several medical and psychosocial complications. A study with 152 treatment-seeking individuals with obesity found that those with binge eating disorder had higher BMIs, more severe levels of depression and obsessive-compulsive symptoms, and stronger feelings of inadequacy and inferiority than those without binge eating disorder [20]. Similarly, bariatric surgery candidates with comorbid binge eating disorder had significantly more mood and anxiety disorders than bariatric surgery candidates without binge eating disorder (27% versus 5% for mood disorders, and 27% versus 8% for anxiety disorders, respectively) [21]. Indeed, 40% of bariatric surgery candidates with comorbid binge eating disorder had a mood or anxiety disorder, with some participants having both a mood and an anxiety disorder [21]. Similarly, gastric bypass surgery candidates with binge eating disorder had more disordered eating attitudes and behaviors, as well as worse physical, emotional and social quality of life, than gastric bypass surgery candidates without binge eating disorder [22]. Not only is binge eating in individuals with obesity associated with poor mental health and poor quality of life, but binge eating can also hinder weight loss in individuals with morbid obesity. For instance, a systematic review found that individuals submitted to bariatric surgery that had clinically significant binge eating before and after the surgery had worse weight loss outcomes than those without pre-surgical binge eating, or than those who stopped binge eating after the surgery [23]. The occurrence of obesity in individuals with eating disorders is also associated with greater mental health complications. For instance, individuals with eating disorders that had had obesity at any stage in their lives had higher eating disorder severity and greater general psychopathology than those with eating disorders that had never had obesity [16]. Finally, obesity with comorbid binge eating can be functionally detrimental. For example, individuals with obesity and comorbid binge eating had greater work-related impairment in productivity than those with obesity only, or binge eating only, or than those of normal weight without binge eating [24]. Thus, individuals with obesity and comorbid eating disorders are at higher risk of medical and psychosocial complications than individuals with only one or the other condition. However, the most appropriate treatment approaches for individuals experiencing these combined conditions is a controversial topic amongst healthcare professionals.

## 3. The Potential Benefits and Harms of Dieting to Lose Weight

There are often theoretical and clinical debates amongst healthcare professionals regarding the most appropriate treatment approaches for individuals with obesity and comorbid eating disorders. The most controversial aspect of this debate relates to potential benefits and harms of dieting to lose weight. Healthcare professionals specializing in obesity often recommend dieting to their patients or clients with overweight or obesity, encouraging them to reduce and then maintain a healthy weight. Conversely, healthcare professionals specializing in eating disorders, especially those working mainly with individuals with anorexia nervosa and bulimia nervosa, are often concerned about the use of diets driven by idealization of thinness [25].

The negative perception that some healthcare professionals may sustain regarding dieting is understandable when one considers that strict dieting is often a core symptom of eating disorders such as anorexia nervosa and bulimia nervosa [11]. For example, the trans-diagnostic cognitive-behavioral model of eating disorders, which is used to guide the “gold standard” treatment for binge eating disorder and bulimia nervosa, namely cognitive behavior therapy—enhanced (CBT-E), shows strict dieting as a central behavioral component in the maintenance of eating disorders [11]. Furthermore, concerns regarding dieting are derived from studies that found relationships between dieting and eating disorder symptoms [26,27,28]. A classic study in this field, namely the Keys’ study, found that young healthy men submitted to prolonged periods of semi-starvation experienced symptoms that were similar to those experienced by people with eating disorders, such as preoccupation with food, binge eating, distress and depression [26]. Additionally, literature reviews of studies including clinical and non-clinical samples suggest that cognitive restraint can make dieters vulnerable to disinhibition and consequent binge eating [27], and that dietary restriction can lead to binge eating, emotional alterations, distractibility and preoccupation with food and eating [28]. Notwithstanding that dieting can be associated with these negative consequences, one literature review concluded that some dietary restriction (i.e., the consumption of certain foods in moderation) may be necessary for individuals with obesity or for those at risk of developing weight-related health problems in order to reduce the health consequences of overweight [28].

Although there are some important concerns regarding the safety of dieting, the relationship between dieting and binge eating is not clear-cut and needs to be further examined. For instance, a study with 166 patients with bulimia nervosa at admission for residential treatment found that a significant proportion of them (43%) were *not* currently dieting to lose weight or to avoid weight gain [29]. Additionally, this study also found that those who were dieting to lose weight reported lower binge eating frequency in comparison to non-dieters [29]. In line with this, a literature review of studies on different levels of dietary restraint, and retrospective and prospective studies examining the effects of dieting on eating behavior, did not find consistent evidence supporting the view that medically supervised dietary restriction exacerbates binge eating disorder [30]. It is possible that other factors besides dieting need to be present in order to increase the risk for eating disorder behaviors. For example, a prospective study with a population-based sample of 1827 adolescents and young adults and a 10-year follow-up period, found that symptoms of depression and low self-esteem in dieters were important elements increasing the risk of binge eating [31]. Moreover, a narrative review concluded that while dieting may contribute to eating disorders, other factors mediate this relationship, namely a family history of eating disorders, mood disorders, problems with substance/alcohol use, personality characteristics, problematic family interactions, and biological vulnerability [32]. Finally, a systematic review showed that clinically supervised severe energy restriction to treat obesity—as in that used with total meal replacement diets such as very low energy diets—mostly did not cause binge eating, and even reduced binge eating in those with pre-treatment binge eating behaviors [33]. These findings are in line with a previous literature review which showed that moderate dietary energy restriction in combination with behavioral weight loss therapy does not seem to induce binge eating in overweight adults without pre-treatment binge eating, and can reduce binge eating in those with pre-treatment binge eating behaviors [34]. Taken together, these findings suggest that the relationship between dieting and binge eating may be significantly influenced by several other variables, e.g., degree of psychological support and medical need for weight loss. Notably, the young men in the Key’s experiment (see above) were not in medical need of weight loss. Whilst they considered themselves extremely well supervised from a medical perspective [26], psychological effects were less well understood and they would go to great lengths to avoid the shame of dismissal from the trial and the stigma of being a “cheater” when they had broken the diet [29]. The complexity of the relationship between dieting to lose weight and eating disorder behaviors and weight stigma potentially contributes to disagreements amongst healthcare professionals regarding the most appropriate treatment approaches for individuals with obesity and comorbid eating disorders.

## 4. Treatments for Obesity with Comorbid Eating Disorders

Previous studies have examined the effects of treating individuals with obesity and comorbid eating disorders with weight loss or eating disorder treatments, alone or in combination [35,36,37,38]. One study investigated the effects of cognitive behavior therapy (CBT), behavioral weight loss therapy, or a sequential approach of CBT followed by behavioral weight loss therapy on body weight and binge eating [38]. This study showed that at 12-month follow-up, 51% of participants submitted to CBT, 36% of those submitted to behavioral weight loss therapy, and 40% of those submitted to CBT followed by behavioral weight loss therapy, had achieved binge eating remission [39]. It also showed that at 12 months, CBT induced significantly greater reduction in binge eating than behavioral weight loss therapy, and that behavioral weight loss therapy induced significantly greater weight loss than CBT [38]. Moreover, participants who exhibited remission from binge eating had significantly greater reductions in BMI compared to participants who did not [38]. Nonetheless, the reduction in BMI induced by behavioral weight loss therapy was relatively small (i.e., −2.1 kg/m^2^) [38], and the combination of CBT with other obesity treatments could potentially induce greater weight loss. In line with this, another study found significant reductions in body weight (average loss of 12 kg) and binge eating at 6 months from treatment commencement in individuals submitted to a diet allowing 7100 kJ (1700 kcal) per day, combined with CBT, sertraline (a serotonin reuptake inhibitor) and topiramate (an anti-convulsant sometimes used in the treatment of obesity) [35].This same study did not show any significant change in body weight or binge eating in the comparison groups of individuals submitted to a 7100 kJ (1700 kcal) per day diet, CBT and sertraline (without topiramate), or in those submitted to nutritional counselling and CBT [35]. We could thus conclude from this study that complex treatments including dietary intervention, CBT and a combination of pharmaceuticals can induce substantial reductions in body weight and binge eating. Another study assessed the addition of CBT to 16 sessions of group behavioral weight loss therapy in 116 women with overweight/obesity and comorbid binge eating disorder [36]. In this study, the serotonin reuptake inhibitor fluoxetine (or placebo) was also investigated. Thus, participants were randomized to one of the following four groups, with all groups receiving the same behavioral weight loss therapy: (1) CBT + fluoxetine; (2) CBT + placebo; (3) fluoxetine; or (4) placebo [36]. This study showed that participants in the groups that included CBT had significantly greater reductions in binge eating frequency, and greater abstinence from binge eating, than participants in groups that did not include CBT [36]. Additionally, this study found that participants reporting abstinence from binge eating at the end of treatment (*n* = 54) exhibited greater weight loss in comparison to those who did not report abstinence from binge eating (*n* = 62) (i.e., a weight loss of 6.2 kg versus weight gain of 0.7 kg, respectively) [36]. From these findings it thus seems that CBT is particularly important for binge eating reduction and abstinence, and that binge eating abstinence may be necessary for successful weight loss. While CBT appears to be an essential element of treatment, another study suggests that there is some flexibility in the types of dietary interventions that can be combined with CBT in order to reduce body weight and binge eating. Indeed, in another study involving 50 individuals with obesity and comorbid binge eating disorder, participants were randomized to CBT + a diet of low energy density, or CBT + general nutrition counselling [37]. This study found that 13 out of the 43 participants that completed their assigned intervention lost at least 5% of their initial body weight, and reduced binge eating episodes by 55–75%, with no significant differences in outcomes between participants in the two groups [37]. Overall, the above-mentioned studies showed that certain combinations of obesity treatments with CBT for eating disorders can induce significant reduction of binge eating and weight loss in individuals with obesity and comorbid eating disorders.

While some scientific studies have examined the effects of combining obesity treatments with eating disorder treatments [35,36,37], most of the current treatment programs for obesity or eating disorders still target only one or the other condition. For example, CBT-E is suitable for use in binge eating disorder, however, it does not encourage weight loss for those with overweight or obesity and comorbid binge eating disorder [11], nor does it result in substantial weight loss [38,39]. In the same way, a recent systematic review and meta-analysis on the effectiveness of treatments for binge eating disorder found that therapist-led CBT induced greater reduction of binge eating frequency in comparison to behavioral weight loss therapy; however, greater body weight reduction was achieved with behavioral weight loss therapy in comparison to CBT [39]. According to this systematic review, at the end of treatment, participants submitted to CBT reduced BMI by an average of 0.41 kg/m^2^ (which is not clinically significant), whereas participants submitted to behavioral weight loss therapy reduced BMI by an average of 2.2 kg/m^2^ (which is clinically significant) [39]. Furthermore, another review of treatments for binge eating disorder concluded that weight loss treatments should be offered only to individuals with obesity that do not engage in binge eating [40]. This contrasts with a study which found that behavioral weight loss therapy, although not designed to thoroughly address eating disorder psychopathology, concomitantly reduced binge eating and body weight in individuals with obesity and comorbid binge eating disorder [38]. Additionally, a systematic review and meta-analyses found a lack of treatments aiming to simultaneously assist weight management and reduce eating disorder behaviors in individuals with obesity and comorbid bulimia nervosa [41]. According to that systematic review and meta-analysis, it is necessary to develop and test integrated treatments for obesity with bulimia nervosa due to the rise in prevalence of individuals with obesity and comorbid bulimia nervosa [41]. In line with this, we have been involved with the development of a new integrated treatment, named HAPIFED (a **H**ealthy **AP**proach to we**I**ght management and **F**ood in **E**ating **D**isorders), which aims to simultaneously aid weight management (i.e., via a moderate to slow rate of weight loss) and to reduce eating disorder behaviors in individuals with obesity and comorbid eating disorders [42]. The feasibility and acceptability of this intervention was shown in a pilot study that used 20 sessions of group multidisciplinary therapy [42], and this treatment is currently under examination in a randomized controlled trial comparing 30 sessions of group therapy of either HAPIFED or CBT-E [43]. Moreover, the effectiveness of HAPIFED in “real world” clinics is also currently been investigated [44].

Integrated treatments, such as HAPIFED, may be particularly attractive to individuals with obesity and comorbid eating disorders. For instance, a systematic review found that only an estimated 23% of individuals with eating disorders sought treatment for their eating disorder, whereas 30–73% of individuals with eating disorders sought treatments for their overweight or obesity (13). This tendency for individuals with eating disorders to seek weight loss treatments instead of eating disorder treatments could potentially be used to facilitate treatment of a significant proportion of individuals with eating disorders that are overweight or have obesity. In addition to potentially improving treatment adherence, integrated treatments for obesity with comorbid eating disorders may also promote greater physical and mental health benefits than treatments focused on only one or the other condition. For instance, individuals with clinically significant binge eating are more susceptible to difficulties achieving a healthy body weight after undergoing a weight loss treatment (i.e., bariatric surgery) in comparison to those without clinically significant binge eating [23]. Therefore, it is important to further investigate integrated treatment options aiming to simultaneously reduce body weight and eating disorder behaviors and to discuss these studies with healthcare professionals working with clients with obesity or eating disorders. This is especially important given that healthcare professionals working in the fields of obesity or eating disorders frequently have different professional backgrounds (i.e., psychological versus medical versus nutritional).

## 5. Conclusions

Individuals with obesity and comorbid eating disorders are at higher risk for several medical and psychosocial complications than individuals with either condition alone. The co-occurrence of obesity with comorbid eating disorders, particularly binge eating disorder, requires attention of healthcare professionals working with clients with either of these conditions. Healthcare professionals specialized in obesity treatment and not addressing eating disorder behaviors of their clients—when these are present—will likely see unsuccessful weight loss interventions in the long-term due to continued binge eating. On the flip side, healthcare professionals specialized in eating disorders and not addressing weight management with their clients with comorbid overweight or obesity may see a reduced interest from their clients, potentially resulting in disengagement from (or lack of engagement at all in) the eating disorder treatment, seeking instead weight loss treatments that do not address their eating disorder behaviors. While these healthcare professionals may sometimes disagree on the potential benefits and harms of recommending weight loss diets to their clients, healthcare professionals working in either of these fields of obesity or eating disorders can acknowledge the common goal of promoting healthy eating behaviors, healthy relationships of their clients with their bodies, and positive personal health goals of weight loss. We contend, it is vital to develop and test innovative medical, psychological, and nutritional treatment options that simultaneously address obesity as well as comorbid eating disorders. Such integrated treatments can potentially induce greater improvements in health—and in more people—than treatments targeting only one or the other condition.
